# Freezing of gait and dementia in parkinsonism: A retrospective case–control study

**DOI:** 10.1002/brb3.1247

**Published:** 2019-05-09

**Authors:** Giovanni Palermo, Daniela Frosini, Andrea Corsi, Martina Giuntini, Sonia Mazzucchi, Eleonora Del Prete, Ubaldo Bonuccelli, Roberto Ceravolo

**Affiliations:** ^1^ Department of Clinical and Experimental Medicine, Unit of Neurology University of Pisa Pisa Italy

**Keywords:** dementia with lewy bodies, freezing of gait, Parkinsonism, Parkinson's disease

## Abstract

**Objectives:**

To support the cognitive model of Freezing of Gait (FoG) we investigated FoG in a cohort of patients with Dementia with Lewy Bodies (DLB).

**Materials and Methods:**

We assessed FoG frequency in 19 DLB patients compared to 19 control PD patients within 2 years from symptom onset and with at least 5 years follow‐up. The two groups were matched by age and motor presentation at onset, severity of parkinsonism and disease duration. The presence and severity of FoG was identified as those with a score of 1 or greater on subitem 14 of the Unified Parkinson's Disease Rating Scale Part II  (UPDRS II).

**Results:**

At T0, 68.4% DLB and 10.5% PD patients experienced FoG ≥1. The prevalence of FoG increased with disease progression (94.7% DLB and 47.3% PD subjects had FoG ≥1 at T5). DLB also showed a more severe FoG (FoG ≥2) than PD (21% vs. 0% at T0 and 52.6% vs. 10.5% at T5), consistently with previous studies reporting FoG prevalence in DLB.

**Conclusion:**

This is the first study looking specifically at FoG in DLB, identifying it as a frequent and early feature of DLB and emphasizing the crucial role of cognitive impairment in the occurrence of this mysterious phenomenon.

## 1 INTRODUCTION

Freezing of Gait (FoG) is an episodic, brief, and unpredictable gait disorder in which patients feel as their feet are glued to the ground with an inability to produce effective steps despite the intention to walk (Nutt et al., 2011). It is a very disabling symptom, correlates with postural instability, interferes with daily life, impairs mobility, and represents one of the major causes of falls. Treatment is a challenging task and the medical management is often ineffective. FoG can be classified on the basis of its response to dopaminergic treatments in FoG‐ON, which is resistant to dopaminergic replacement therapy, and FoG‐OFF (the most common), which is relieved by dopaminergic medication. Levodopa can sometimes induce or even worsen FoG. This suggests that FoG could recognize a neuropathology that exceeds dopaminergic circuits. Furthermore, FoG can be distinguished on the basis of its appearance: FoG in the middle of motion, “start hesitation,” “turning hesitation,” FoG when approaching a destination or an obstacle, or FoG when walking in narrow spaces (Schaafsma et al., 2003); each subtype of FoG could imply different pathophysiologic substrates. A number of risk factors have been identified: male gender, longer disease duration, and longer duration of levodopa therapy, symptoms severity, motor complications, a postural instability/gait disorder phenotype (PIGD) at onset, cognitive decline, apathy, and depression (Macht et al., 2007).

FoG is common in Parkinson's disease (PD), with prevalence of about 7% in early PD stages, to 80%–90% in Hoehn and Yahr stage 4, but it occurs even more frequently in patients with atypical parkinsonisms (Giladi, Kao, & Fahn, 1997). It can be seen early in the course of PD, even prior to anti‐parkinsonian treatment (Giladi, McDermott et al., 2001), but other parkinsonian syndromes should be suspected if FoG is a presenting sign (e.g., progressive supranuclear palsy, vascular parkinsonism, and normal pressure hydrocephalus).

There are only few reports on FoG in DLB without any cue to the regions potentially involved in the pathophysiology of FoG (Gibb, Esiri, & Lees, 1987; Hely et al., 1996).

Dementia with Lewy bodies (DLB) is an atypical parkinsonian syndrome with the central feature of a progressive cognitive decline. The pathogenesis of FoG is not entirely understood but disruptions in cortical networks have been implicated and cognitive dysfunction is probably one of the main factors contributing to the manifestation of FoG (Heremans et al., 2013). Nevertheless, a model that universally links FoG and dementia has not yet been established and it is currently not known what is the neuroanatomical substrate of FoG.

The study of FoG in DLB could provide an avenue for the further characterization of this phenomenon. Our goal was to investigate the epidemiology of FoG in DLB, as a model of disease with an early relevant cortical pathology.

## 2 MATERIALS AND METHODS

Nineteen PD patients (11F and 8M, aged 57‐79) and 19 DLB patients (9F and 10M, aged 59‐81), with at least 5 years follow‐up, seen since 2008 in the Movement Disorders Clinic of the Neurology Unit, Department of Clinical and Experimental Medicine, University of Pisa, formed the basis for this retrospective study aiming at evaluating differences in FoG prevalence between PD and DLB. Accepted criteria were used to diagnose idiopathic PD (Gelb, Oliver, & Gilman, 1999) and DLB (McKeith et al., 2005). The diagnosis was confirmed by the presence of a typical pattern of nigrostriatal dopaminergic denervation on DaTSCAN imaging for both PD and DLB. At the time of first observation (T0) all DLB patients exhibited parkinsonism and the clinical presentation of parkinsonism was quite similar in PD and DLB subgroups (Table 1). According to case–control study, we selected PD and DLB patients matching them with respect to gender, age and motor presentation at onset (tremor dominant, rigid‐akinetic and mixed phenotypes), disease severity (according to UPDRS III) and disease duration (months) (Table 1). All subjects were evaluated in a standardized fashion over a five‐year period. We collected patient's medical documentation of their regular yearly assessments from T0 to T5. Patients were enrolled if the first evaluation (T0) was done within 2 years from disease onset to capture FoG in the early stages of disease. Motor function was evaluated with the Hoehn and Yahr scale (H&Y) and part III of the Unified Parkinson's disease Rating Scale (UPDRS). The presence of FoG was assessed on clinical examination and on patient history using the five‐point (from 0 to 4) FoG subtest (subitem 14) of the UPDRS part II. Subjects were then categorized in FoG = 1 and FoG ≥2 to measure also the different severity of this symptom. The study was approved by the local ethics committee.

**Table 1:** Baseline demographics for DLB and PD patients

	DLB	PD	*p* value
Gender, F/M	9/10	11/8	0.74^a^
Age of onset, mean, (range), years	71.89 (59−81)	68.84 (57−79)	0.13^b^
Disease duration (T0), mean, (*SD*), months	10.6 (5.9)	6.6 (6.5)	0.06^b^
UPDRS III subtotal (T0), mean, (range) (normal range 0−108)	27.47 (7−48)	27.05 (8−39)	0.88^b^
Motor presentation at onset	1 TD, 13 R‐A, 5 mixed	1 TD, 13 R‐A, 5 mixed	
MMSE (T0), mean, (range)	19.7 (11.7−27.3)	26.7 (23−30)	0.0007^b^

TD: tremor dominant; R‐A: rigid‐akinetic.

a Fisher exact test.

b Student's *t* test.

### 2.1 Statistical analysis

The occurrence of FoG was compared at different times in DLB and PD patients using Fisher’s exact test (*p* < 0.05 was set as criterion for statistical significance).

## 3 RESULTS

To determine if PD and DLB patients were similar with respect to age, disease duration, and disease severity, we compared the means of two groups using the Student's *t* test while the Fisher's exact test was applied to exclude a gender difference. As we expected, the mean MMSE score was significantly different between the two populations (*p* = 0.0007 from Student's *t* test) (Table 1).

Thirteen of the 19 DLB patients (68.4%) experienced FoG at T0 (mean disease duration ± *SD*: 10.6 ± 5.9 months). At the same time, in PD group at T0 (mean disease duration ± *SD*: 6.6 ± 6.5 months), only 2 of the 19 (10.5%) had FoG. In both populations, the prevalence of FoG increases with disease progression showing a relentless tendency to develop FoG in DLB (Table 2). The total daily dose of levodopa was similar at first (*p* = 0.11) and last evaluation (*p* = 0.34, Tables 3 and 4). Most of DLB patients (89.5%, 17 of 19) suffered FoG after 3 years (T3), and 94.7% (18 of 19) had FoG at T5; amongst PD patients FoG was identified in 7 (36.8%) and in nine (47.3%) of the 19 patients at T3 and T5 respectively. FoG was not only more common in DLB than PD, but it occurred more severe (FoG ≥2) in DLB population (four of the 19 patients at T0, 21%), while none of PD patients showed FoG ≥2 at the same time, even though the difference observed was not statistically different (*p* = 0,10). A more severe FoG (FoG ≥2) confirmed its high occurrence in DLB population also in the follow‐up with more than half of the patients who developed FoG at T5 (52.6%, vs. 10.5% of PD patients) (Table 5). Four DLB patients (21.05%) were treated with antipsychotic medications at T0 and the number of DLB patients undergoing treatment with these drugs was higher at T5 (11 patients, 57.9%); all these patients used atypical antipsychotics (Quetiapine or Clozapine). 21.05% and 84.2% of DLB patients were treated with cholinesterase inhibitors at T0 and T5 respectively (one DLB patient received Memantine at the first and last evaluation). Neither at baseline nor at follow‐up visits PD patients did receive antipsychotic or cholinesterase inhibitors medications.

**Table 2:** Five‐year prevalence of FoG in DLB and PD patients

		FoG+	FoG‐
T0	DLB	13 (68.4%)	6 (31.6%)
	PD	2 (10.5%)	17 (89.5%)
	*p* value	0.0006	
T1	DLB	16 (84.2%)	3 (15.8%)
	PD	2 (10.5%)	17 (89.5%)
	*p* value	0.0001	
T2	DLB	16 (84.2%)	3 (15.8%)
	PD	5 (26.3%)	14 (73.7%)
	*p* value	0.0008	
T3	DLB	17 (89.5%)	2 (10.5%)
	PD	7 (36.8%)	12 (63.2%)
	*p* value	0.0019	
T4	DLB	17 (89.5%)	2 (10.5%)
	PD	8 (42.1%)	11 (57.9%)
	*p* value	0.0051	
T5	DLB	18 (94.7%)	1 (5.3%)
	PD	9 (47.3%)	10 (52.6%)
	*p* value	0.003	

**Table 3:** Detailed therapy DLB versus PD patients (T0)

	DLB	PD
Levodopa/Carbidopa	11/19 (57.9%)	11/19 (57.9%)
Melevodopa	1/19 (5.3%)	0/19 (0%)
Levodopa/Carbidopa/Entacapone	0/19 (0%)	0/19 (0%)
Levodopa/Benserazide	1/19 (5.3%)	0/19 (0%)
Pramipexole	1/19 (5.3%)	2/19 (10.5%)
Ropinirole	0/19 (0%)	1/19 (5.3%)
Rasagiline	0/19 (0%)	1/19 (5.3%)
Rotigotine	0/19 (0%)	0/19 (0%)
Selegiline	2/19 (10.5%)	0/19 (0%)
Amantadine	1/19 (5.3%)	0/19 (0%)
Levodopa Equivalent Daily Dose	158.2 mg/die	98.2 mg/die

**Table 4:** Detailed therapy DLB versus PD patients (T5)

	DLB	PD
Levodopa/Carbidopa	14/19 (73.7%)	14/19 (73.7%)
Melevodopa	1/19 (5.3%)	5/19 (26.3%)
Levodopa/Carbidopa/Entacapone	0/19 (0%)	1/19 (5.3%)
Levodopa/Benserazide	1/19 (5.3%)	2/19 (10.5%)
Pramipexole	0/19 (0%)	5/19 (26.3%)
Ropinirole	0/19 (0%)	3/19 (15.8%)
Rasagiline	0/19 (0%)	3/19 (15.8%)
Rotigotine	0/19 (0%)	3/19 (15.8%)
Selegiline	0/19 (0%)	0/19 (0%)
Amantadine	1/19 (5.3%)	2/19 (10.5%)
Levodopa Equivalent Dose	565.8 mg/die	654 mg/die

**Table 5:** Prevalence of mild to severe FoG (FoG ≥2) in DLB and PD patients

		FoG ≥2	NO FoG ≥2
T0	DLB	4 (21%)	15 (78.9%)
	PD	0 (0%)	19 (100%)
	*p* value	0.10	
T1	DLB	6 (31.6%)	13 (68.4%)
	PD	1 (5.3%)	18 (94.7%)
	*p* value	0.089	
T2	DLB	8 (42.1%)	11 (57.9%)
	PD	1 (5.3%)	18 (94.7%)
	*p* value	0.018	
T3	DLB	9 (47.4%)	10 (52.6%)
	PD	1 (5.3%)	18 (94.7%)
	*p* value	0.0078	
T4	DLB	9 (47.4%)	10 (52.6%)
	PD	2 (10.5%)	17 (89.5%)
	*p* value	0.029	
T5	DLB	10 (52.6%)	9 (47.4%)
	PD	2 (10.5%)	17 (89.5%)
	*p* value	0.0128	

## 4 DISCUSSION

We found that FoG is a frequent feature of DLB supporting the link between this disorder and dementia. Moreover we confirm that FoG may be an early sign in the course of conditions other than PD, heralding a diagnosis of DLB. Freezing is a complex and disabling motor disturbance that affects severely patient's walking and quality of life (Moore, Peretz, & Giladi, 2005). FoG is common in PD, where it is one of the most severe late symptoms (Giladi, Treves et al., 2001). However, FoG has been reported also at the early stages of PD (Giladi, McDermott et al., 2001). This suggests that a severe dopamine depletion may be not essential to FoG. To date, the nature of FoG and its natural history remain controversial. Although few studies have been done to assess FoG in atypical parkinsonian syndromes, we know that FoG is a frequent presenting sign in atypical parkinsonisms and other conditions such as normal pressure hydrocephalus and vascular parkinsonism supporting further the role of nondopaminergic networks (Factor, 2008). In a retrospective survey, Giladi and co‐workers were the first to report the large prevalence of FoG in populations of 347 patients with degenerative parkinsonisms other than PD. Early dementia was inclusive criteria and they observed an association between FoG and dementia but DLB was not mentioned (Giladi et al., 1997). A similar retrospective analysis, in autopsy confirmed atypical parkinsonian disorders, including DLB patients with a median survival of 58 months, was conducted in a multicenter clinicopathologic study of 66 subjects (Muller et al., 2002). The authors confirmed the remarkable frequency of FoG in parkinsonisms and the increased probability to have FoG with the disease duration. In DLB group, FoG was recorded in three out of the 14 patients (21%) at first visit (that corresponded for all the patient to 36 months from symptom onset) and in seven patients (54%) at the second neurological visit (70.5 months from symptom onset).  In his overview of FoG in atypical parkinsonian conditions, Factor confirmed the frequent occurrence of FoG in these entities, such as DLB, with over 50% of patients reporting it in the advanced disease (Factor, 2008). Our data support an even larger prevalence both in early and advanced disease stages of DLB, probably because we included also milder forms of FoG. In this respect, the prevalence of more severe freezing (>2) is quite similar between ours and previous studies. Our study is the first that looked specifically at the epidemiology of FoG in DLB, adopting a case–control design with PD patients to correct potential contributing factor to FoG such as age and clinical presentation at onset, longer disease duration and greater disease severity. We found that FoG is a consistent and early feature of DLB, pointing to the possibility that FoG represents a red flag of this clinicopathologic entity; furthermore, it highlights the specific relationship between FoG and dementia. Dementia and axial motor involvement including FoG dominate the clinical picture of late stages of PD; it is in keeping with extranigral pathology and with the later involvement of nondopaminergic neurotransmitter system, such as the serotonergic, noradrenergic and cholinergic circuits (Coelho & Ferreira, 2012). Increasing evidence from autopsy and imaging studies indicates that the cholinergic system has widespread influence on a number of nonmotor symptoms in PD, including cognition, gait, and postural instability (Bohnen et al., 2006, 2009). In this respect, we should take into account that FoG was more common in our DLB patients than in PD cases, despite the presence of treatment with Cholinesterase inhibitors in most of DLB patients.  A large amount of evidence has shown that FoG and dementia are two related phenomena; it is well‐described the independent contribution of cognitive function to FoG (Giladi & Hausdorff, 2006; Vercruysse et al., 2012). FoG has been associated with a dysexecutive syndrome and with visuospatial abnormalities in PD that are the typical findings in the cognitive profile of both PDD and DLB patients. DLB and PDD are believed to be phenotypes of a disease spectrum in which the central clinical manifestations are parkinsonism, dementia, and hallucinations (Berg et al., 2014). If dementia is one of the determinants of FoG, we expected that DLB patients showed a high occurrence of FoG‐episodes compared with PD patients without dementia. Our results, once excluded the possible interference of motor disease severity and duration, indicates that FoG is probably associated with the more severe cognitive involvement of DLB, supporting the cognitive model of FoG (Nieuwboer & Giladi, 2013). Indeed, taking into account the difficulty in comparing patients with different diseases and the limits of the retrospective nature of the study, the presence of dementia remains the only clinical feature differentiating the two groups.  It would be interesting to compare the neuropsychological profile of DLB patients with and without FoG at disease onset in order to evaluate a cognitive profile more prone to be associated with FoG, but this is a limitation to our study due its retrospective nature. Furthermore, we could not characterize FoG to investigate even subtle differences in its phenomenology between PD and DLB, with respect to the time of onset and the response to levodopa, distinguishing an early FoG, with a changeable response to dopaminergic therapy, and a late FoG in advanced disease that is poorly affected by medication (Espay et al., 2012). Another important caveat of our study could be that diagnosis and measurement of FoG were made through patient self‐report and on UPDRS‐ADL item 14. It could be unreliable;l however it has been demonstrated that this item correlates strongly with specific FOG questionnaires developed more recently (Giladi et al., 2009).

## 5 CONCLUSIONS

This is the first study looking specifically at phenomenon FoG in DLB, identifying FoG as a frequent and early feature of this atypical parkinsonism. Currently, the anatomic basis for FoG remains unclear and a variety of brain regions have been identified (Fasano, Herman, Tessitore, Strafella, & Bohnen, 2015; Snijders et al., 2016). By investigating FoG in DLB, a parkinsonism characterized by dementia, our findings of a greater prevalence in such patients compared to matched PD patients, emphasize the crucial role of cognitive impairment in the occurrence of FoG and can contribute to the understanding of the pathogenesis of freezing of gait. A longitudinal prospective study is warranted to confirm these preliminary evidences.

## CONFLICT OF INTEREST

The authors declare that they have no conflict of interest.
